# Complete coverage by examinations: relationship to colorectal cancer burden and the COVID-19: Ondrej Ngo

**DOI:** 10.1093/eurpub/ckac131.031

**Published:** 2022-10-25

**Authors:** O Ngo, J Kouril, S Suchanek, L Dusek, B Seifert, M Zavoral, O Majek

**Affiliations:** 1 Institute of Health Information and Statistics, Prague, Czechia; 2 Institute of Biostatistics and Analyses, Faculty of Medicine, Masaryk University, Brno, Czechia; 3 Department of Medicine, 1st Faculty of Medicine, Military University Hospital, Prague, Czechia; 4 Institute of General Practice, 1st Faculty of Medicine, Charles University, Prague, Czechia

## Abstract

**Background:**

Colorectal cancer (CRC) is among the most common cancers and cancer causes of death worldwide. CRC screening and early detection is essential to reduce CRC incidence and mortality. CRC screening has been initiated in the Czech Republic in 2000 for persons over 50 and currently offers a faecal occult blood test (FOBT) or screening colonoscopy (CS). The aim of our study was to present complete coverage by examinations in relation to the trends in CRC burden and impact of COVID-19.

**Methods:**

We defined the complete coverage by examinations as the proportion of persons aged over 50 undergoing examination with CRC early detection potential (FOBT or CS for any indication) during past 3 years. Standardized incidence and mortality rates were used to assess epidemiological trends. The impact of COVID-19 was assessed for 2020 and 2021 by comparing the volume of examinations with 2019. We used national health registries (National Registry of Reimbursed Health Services, Czech National Cancer Registry) as the source of data.

**Results:**

Complete coverage was increasing over time and reached around 50% in recent years (target population is more than 4 million persons, most of the performed examinations were screening FOBT). However, coverage has decreased to 47.9% in 2020. In 2020 and 2021, the number of tests performed decreased by 16.9% and 5.5%, respectively, compared to 2019. CRC incidence and mortality rates have decreased by more than 20% and almost 30%, respectively, in the last decade.

**Conclusions:**

Complete coverage has reached a satisfactory level and has likely a positive impact on the epidemiological trends. However, further action is needed to increase coverage, recently affected by COVID-19 pandemic, when non-acute health care may have been neglected.

**Key messages:**

• The long-term high level of coverage by examinations likely has a positive impact on CRC burden.

• The observed decrease in coverage caused by COVID-19 needs to be appropriately compensated.

